# Phenolic Compounds Contained in Little-known Wild Fruits as Antiadhesive Agents Against the Beverage-Spoiling Bacteria *Asaia* spp.

**DOI:** 10.3390/molecules22081256

**Published:** 2017-07-28

**Authors:** Hubert Antolak, Agata Czyzowska, Marijana Sakač, Aleksandra Mišan, Olivera Đuragić, Dorota Kregiel

**Affiliations:** 1 Institute of Fermentation Technology and Microbiology, Lodz University of Technology, Wolczanska 171/173, 90-924 Lodz, Poland; agata.czyzowska@p.lodz.pl (A.C.); dorota.kregiel@p.lodz.pl (D.K.); 2 Institute of Food Technology Novi Sad, Bulevar cara Lazara 1, 21000 Novi Sad, Serbia; marijana.sakac@fins.uns.ac.rs (M.S.); aleksandra.misan@fins.uns.ac.rs (A.M.); olivera.djuragic@fins.uns.ac.rs (O.Đ.)

**Keywords:** *Asaia* spp., fruit juices, berry juices, polyphenols, anti-adhesion

## Abstract

The aim of the study was to evaluate antioxidant activity and total phenolic content of juice from three different types of fruits: elderberry (*Sambucus* *nigra*), lingonberry (*Vaccinium* *vitis-idaea*) and cornelian cherry (*Cornus* *mas*), and their action against adhesion of bacterial strains of *Asaia* *lannensis* and *Asaia* *bogorensis* isolated from spoiled soft drinks. The antioxidant profiles were determined by total antioxidant capacity (2,2-diphenyl-1-picrylhydrazyl, DPPH), and ferric-reducing antioxidant power (FRAP). Additionally, total polyphenol content (TPC) was investigated. Chemical compositions of juices were tested using the chromatographic techniques: high-performance liquid chromatography (HPLC) and liquid chromatography–mass spectrometry (LC-MS). Adhesion properties of *Asaia* spp. cells to various abiotic materials were evaluated by luminometry, plate count and fluorescence microscopy. Antioxidant activity of fruit juices expressed as inhibitory concentration (IC_50_) ranged from 0.042 ± 0.001 (cornelian cherry) to 0.021 ± 0.001 g/mL (elderberry). TPC ranged from 8.02 ± 0.027 (elderberry) to 2.33 ± 0.013 mg/mL (cornelian cherry). Cyanidin-3-sambubioside-5-glucoside, cyanidin-3-glucoside, and cyanidin-3-sambubioside were detected as the major anthocyanins and caffeic, cinnamic, gallic, protocatechuic, and *p*-coumaric acids as the major phenolic acids. A significant linear correlation was noted between TPC and antioxidant capacity. In the presence of fruit juices a significant decrease of bacterial adhesion from 74% (elderberry) to 67% (lingonberry) was observed. The high phenolic content indicated that these compounds may contribute to the reduction of *Asaia* spp. adhesion.

## 1. Introduction

The presence of spoilage microorganisms in production lines increases the risk of cross-contamination of products and in the case of certain groups of microorganisms it can pose a significant risk to public health [1]. Representative microorganisms increasingly isolated from functional drinks are the Gram-negative acetic acid bacteria *Asaia* spp. Numerous studies on *Asaia* spp. and their presence in non-alcoholic beverages are described in the literature [2,3]. It was noted that the growth of these bacteria causes significant changes in organoleptic qualities of final products, such as turbidity and flock formation. Furthermore, *Asaia* strains are characterized by strong adhesive abilities with respect to materials in contact with food. Consequently, the adhesion and proliferation of these bacteria on solid surfaces leads to the formation of biofilms which can be potential sources of product contamination [2]. In addition, *Asaia* spp. are considered to be opportunistic pathogens. They can cause infections in people with immunodeficiency, such as pediatric patients, children in general, and patients with a history of intravenous drug abuse. Moreover, *Asaia* strains are characterized by high resistance to chemical preservatives commonly used to improve the microbiological stability of food, such as benzoates, sorbates and dimethyldicarbonate. In general, in the case of microbial cells forming biofilms, increased resistance to commonly used disinfectants such as quaternary ammonium compounds, peracetic acid and hydrogen peroxide is observed [4,5]. Therefore, there is a growing demand for effective alternatives to the chemicals used against these bacteria [6].

Medicinal plants have historically proven value as natural sources of molecules with therapeutic potential. However, in the past decades, the pharmaceutical industry has focused mainly on synthetic compounds as sources of drug discovery [7]. On the other hand, social interest in old medicinal plants is growing, and the global market of herbal medicines stands at over 60 billion of United States dollars annually, generating increasing interest and publicity. The importance of plants results from the fact that bioactive compounds found in the wild cannot be reproduced in the laboratory. As a result, it is estimated that about 60% of antimicrobial drugs discovered in the past few decades are of natural origin [8]. The use of plants with antimicrobial activities is particularly important in the food industry. Many research projects aim to identify and characterize natural products by the combined and synergistic use of computational techniques, ethnopharmacological knowledge, chemistry, and a broad range of cell-based models [9].

Among the plant origin sources of bioactive compounds characterized by antimicrobial and antiadhesive activities, berries are one of the most prominent. Recent reviews showed that phenolic compounds from berries show antimicrobial activity against fungi, viruses, and bacteria, including methicillin-resistant *Staphylococcus aureus* (MRSA) strains [10]. In vitro studies showed that particular berries, like cranberry (*Vaccinium macrocarpon*), cloudberry (*Rubus chamaemorus*), bilberry (*Vaccinium myrtillus*) and strawberry are characterized by antimicrobial action against pathogens belonging to *Escherichia*, *Salmonella*, *Staphylococcus*, *Helicobacter*, *Clostridium* and *Campylobacter* genera or their inflammatory agents, such as endotoxin lipopolysaccharide (LPS) [11,12,13]. Effective activity of fruit juices was also noted against yeasts and molds such as *Candida krusei*, *Candida albicans*, *Trichophyton tonsurans*, and *Aspergillus fumigatus* [14]. These fruits are rich sources of anthocyanins, phenolic acids, flavanols, flavonols, and tannins, with health promoting actions, and have been used for centuries in folk medicine as natural remedies for many diseases [15]. In addition, these compounds show high antioxidant capacity and anti-ulcer, anti-inflammatory, anti-cancer and anti-microbial properties [16,17]. Additionally fruit juices can be used as natural food colorings and aromas in the food industry, which excludes the need to use synthetic equivalents.

While some fruits are common on supermarket shelves around the world, there are many other wild-growing fruit that are not so well-known. These include elderberry (*Sambucus nigra*) and lingonberry (*Vaccinium vitis-idaea*), as well as cornelian cherry (*Cornus mas*). Their common feature is that they grow as a perennial wild plant in Europe and they are known in traditional folk medicine. *Sambucus nigra* fruits and flowers have been used in traditional medicine internally for treatment of disorders of the respiratory and gastrointestinal tracts, mouth, skin, and for viral infections, fever, colds, and influenza [18]. *V. vitis-idaea* has a long history of use as an anti-hemorrhagic, antiseptic and anti-urogenital infection agent. *Cornus mas* is considered as the least known in Europe but this uncommon fruit is rich in vitamin C, and can be used to fight cold and flu [19]. Early studies of these wild-growing fruits focused mainly on their direct antimicrobial potential against pathogenic bacteria [20]. However, much less attention has been paid to activities against spoilage microflora in production lines in the beverage industry. Therefore, the aim of our study was to characterize bioactive components of these three juices and evaluate their antioxidant activity in vitro, as well as their action against adhesion of *Asaia* spp. bacteria isolated from spoiled soft drinks.

## 2. Results and Discussion

### 2.1. Carbohydrate Content

Each type of fruit juice was analyzed for two of the most abundant dietary sugars: glucose and fructose, by spectrophotometric method using enzymatic assay kits specific for these carbohydrates. This method is characterized by high sensitivity, with lower limit of detection equaling 0.332 mg/L. Sugar content varied depending on the type of fruit. All tested fruit juices contained fructose, but the highest content of this monosaccharide was found in the juice of cornelian cherry (5.56 ± 0.061 g/100 mL) (Table 1). However, this juice contained the lowest glucose content, equaling 2.97 ± 0.046 g/100 mL. The lowest fructose content (3.29 ± 0.015 g/100 mL) was noted for elderberry juice, and this value was similar to the elderberry glucose content (3.19 ± 0.022 g/100 mL). The results of our research are in line with the available literature. Carbohydrate content in elderberry is 18%, 11.5% of which are monosaccharides. Over 95% of these monosaccharides are fructose and glucose, present in similar amounts [18]. According to Veberic et al. (2009), fresh elderberry fruits contain fructose and glucose, and their amounts range from 3.39 ± 0.093 to 5.25 ± 0.143 and from 3.333 ± 0.067 to 5.23 ± 0.053 g/100 g, respectively [21]. The reducing sugar content in cornelian cherry ranges from 5.2 to 12.00 g/100 g of fresh weight, with an average value of 8.1 ± 1.6 g/100 g. In a manner similar to *S. nigra* and *C. mas*, the main sugars in lingonberry juice are glucose and fructose, with contents of 4.3 g/100 mL and 4.4 g/100 mL, respectively [22]. The differences in sugars content can be attributed primarily to the genotype of the plants, as well as geographical location and prevailing climatic conditions [23].

### 2.2. Antioxidant Capacity and Total Phenolic Content

Generally, several assays have been frequently used to estimate antioxidant capacity in fruits and their products, such as 2,2-azinobis(3-ethyl-benzothiazoline-6-sulfonic acid) (ABTS), 2,2-diphenyl-1-picrylhydrazyl (DPPH), and ferric-reducing antioxidant power (FRAP). In addition, in antioxidant capacity assays, peroxyl (ORAC), hydroxyl (HORAC), superoxide anion (SORAC), peroxynitrite (NORAC), and singlet oxygen (SOAC) radicals/oxidants are used [24]. However, FRAP and DPPH, which are based generally on a single-electron transfer (SET) mechanisms and measure the ability of an antioxidant to transfer one electron to reduce a compound, are still the most commonly used. The DPPH test measures the ability to scavenge free radicals, while the FRAP assay quantifies the total concentration of redox-active compounds. Both tests are simple, relatively rapid, reproducible and do not require specialized equipment, and thus can be used for assessing antioxidant activity in foods and plant extracts.

The results of DPPH test obtained in our study indicate that cornelian cherry juice showed the strongest antioxidant properties, reported as inhibitory concentration, (IC_50_ = 0.045 ± 0.001 g/mL), while the lowest capacity was obtained for elderberry juice (IC_50_ = 0.072 ± 0.001 g/mL). It has been documented that the water extract of elderberry has lower DPPH radical scavenging capacity than that of bilberry or chokeberry, but is comparable to raspberry fruit extract [25]. On the other hand, in a study by Jakobek et al. (2007) elderberry juice showed stronger antioxidant activity than black currant, red raspberry, sour cherry, and sweet cherry, as well as strawberry juices [26]. It is worth noting that wild growing *C. mas* shows stronger DPPH radical scavenging ability than the cultivated forms [27]. According to Georgieva and co-workers (2016), lingonberry showed lower DPPH capacity than strawberry, raspberry and bilberry, while Tarko et al. (2015) noted that beverages supplemented with 2% lingonberry juice exhibited lower antioxidant activity than products enriched with 2% cornelian cherry juice [28,29].

The FRAP test results obtained in our study indicate that the elderberry juice (IC_50_ = 0.021 ± 0.001 g/mL) was characterized by the strongest activity, followed by lingonberry juice (IC_50_ = 0.030 ± 0.002 g/mL). The results of DDPH and FRAP assays obtained in our study strongly correlate with the results of total polyphenol content (TPC) tests. For FRAP method, the R coefficient amounted to 0.9896 (y = −0.0037x + 0.0496) while for DPPH this value was 0.9917 (y = 0.0048x + 0.0327). TPC was the highest in elderberry juice, and the lowest in cornelian cherry juice (Table 1). As it had the highest TPC, elderberry juice was characterized with the strongest antioxidant activity as measured using FRAP assay. According to Tarko and co-workers (2015) TPC in lingonberry juice is comparable to cornelian cherry and represents 51 ± 1.1 and 51 ± 1.0 mg catechin/100 mL, respectively [29]. In the work of Šamec and Piljac-Žegarac (2011) cornelian juice was characterized by high content of phenols reaching the level of 501.58 ± 10.11 mg of gallic acid equivalents (GAE)/100 g, thereby providing a richer source of phenolic compounds than red (147.39 ± 2.42 mg GAE/100 g) and white (60.12 ± 3.05 mg GAE/100 g) grapes [30]. These results agree with those obtained by Moldovan and co-workers (2016), who noted that the TPC for fresh *C. mas* fruits extract was 489.94 ± 17.88 mg GAE/100 g [31]. However, the *C. mas* juice tested in our study (recalculating 233 ± 1.00 mg GAE/100 mL) was characterized by lower polyphenol content than that presented by the other authors. Again, these differences may arise not only from geographic determinants, but primarily from plant genotypes.

According to a review of the literature conducted by Nile and Park (2014) elderberry fruits are one of the richest sources of phenolic compounds among berries, with TPC 104 mg GAE/g, while Sidor and Gramza-Michałowska (2015) have reported that this value ranges from 3.6 mg GAE/g to 19.5 mg GAE/g [18,32]. On the other hand, Silva et al. (2017) have shown that elderberry contains approximately 1.19 g GAE/100 g, which is slightly higher than the values obtained in our study [33]. Differences in the obtained values may be due to many factors. For example, climate conditions and fruit variety as well as processing methods (heating, filtration, crushing), and storage conditions (air, temperature) can cause changes in the composition of (poly)phenols [30,34]. It is also worth noting that the stability of anthocyanins from elderberry is higher in the presence of glucose, but significantly lower in the presence of fructose [18]. Furthermore, the antioxidant capacity of biomolecules is significantly influenced by the structure. The activity may be attributed to the enhanced stabilization of the radical state during electron transfer when assayed relative to compounds that lack the orthodiphenolic structure. The addition of a glycoside residue, such as glucose, at position 3 on the C-ring or methylation of the 3′ and/or 5′ hydroxyl group on the anthocyanidin B-ring, has been shown to reduce the antioxidant capacity and radical scavenging activity [35].

### 2.3. Phenolic Profiles

The tested fruit juices are rich source of phenolic acids (PAs) as well as flavonols, flavanols and anthocyanins (Table 2). The presence of these groups of compounds was detected by high-performance liquid chromatography (HPLC) and liquid chromatography–mass spectrometry (LC-MS), therefore additional compounds were also reported (Table 3). In the cornelian cherry the highest content among phenolic acids was obtained for gallic acid (2.025 ± 0.314 µg/mL), while caffeic acid was the major acid for elderberry juice (2.603 ± 0.313 µg/mL). However, despite the greatest variety of compounds in lingonberry juice, the extract contained the lowest amount of phenolic acids.

Our results for cornelian cherry agreed with those obtained by Moldovan et al. (2016) who found that these fruits contain mainly chlorogenic and caffeic acids [31]. However, in their article the authors pointed that the main phenolic acid in the fruit is ellagic acid. Deng et al. (2013) have noted the presence of chlorogenic and gallic acids in *Cornus mas* fruits [36]. On the other hand, Radovanović et al. (2013) found gallic, *p*-coumaric and caffeic acids to be major phenolic acids in these fruits [37]. The lingonberry juice analysed in our research contained protocatechuic acid as a major phenolic acid, with a concentration of 0.497 ± 0.087 µg/mL. Our results on the PAs in the lingonberry juice are in line with those in available literature. In the work of Häkkinen (1999), the most abundant phenolic acids in lingonberry were *p*-coumaric acid (19.9%), ferulic acid (7.0%), caffeic acid (2.6%), hydroxy-benzoic acid (2.1%) and ellagic acid (1.1%) [38]. On the other hand, Mattila and co-workers (2006) found that main PAs were protocatechuic, vanillic, cinnamic, and gallic acids [39]. Subsequently, results of chemical analysis of elderberry juice showed that caffeic acid is the major acid from this group of phenolic compounds. A study conducted by Tarko et al. (2017) noted the presence of caffeic acid in elderberry while Lee and Finn (2007) found cinnamic and chlorogenic to be the main PAs in *S. nigra* [40,41]. Furthermore, Mikulic-Petkovsek et al. (2015) described how different genotypes of elderberry contained different derivatives of coumaric, caffeic and cinnamic acids [42]. Although we did not detect cinnamic acid in our studies using HPLC nor LC-MS techniques, the latter method showed that the juice of elderberry contains chlorogenic acid.

**Figure 1:**
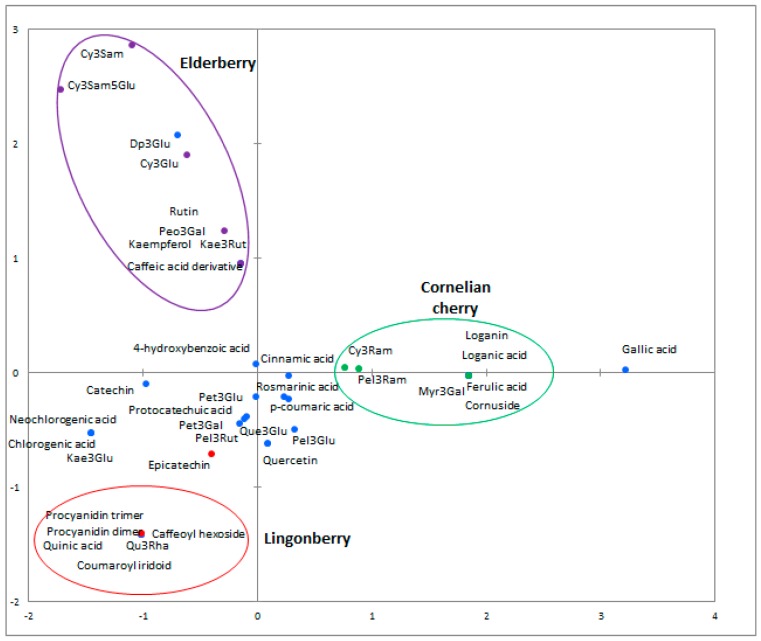
Principal component analysis (PCA) of chemical components identified using HPLC and LC-MS methods. The compounds characteristic for elderberry are marked in purple, lingonberry in red, and cornelian cherry in green. Blue markers correspond to compounds that are common to tested juices.

Other groups of phenolic compounds occurring in fruits are anthocyanins, flavonols, and flavanols. They are responsible for the attractive red, orange, blue, purple and even black colour of fruit. Our results of HPLC analysis showed that only catechin, epicatechin and rutin are present among flavonols and flavanols. On the other hand, the results indicate the presence of delphinidin, cyanidin, petunidin and pelargonidin derivatives. Elderberry was characterized by a variety of anthocyanins and their high concentration. The highest content was noted for cyanidin-3-glucoside, cyanidin-3-sambubioside and cyanidin-3-sambubioside-5-glucoside, the contents were 3.738 ± 0.147 µg/mL, 3.143 ± 0.262 µg/mL and 2.260 ± 0.219 µg/mL, respectively. It is worth noting that cyanidin-3-sambubioside and cyanidin-3-sambubioside-5-glucoside were identified only in *S. nigra* juice (Figure 1).

These results are in accordance with those obtained by other researchers. For example, Silva et al. (2017) noted that three main anthocyanidins in elderberry were cyanidin-3-glucoside, cyanidin-3-sambubioside and cyanidin-3-sambubioside-5-glucoside [33]. However, in their studies the concentrations of these compounds reached higher levels, amounting to 4.27 ± 0.52 g/100 g, 5.59 ± 0.63 g/100 g and 1.79 ± 0.45 g/100 g of dried weight. Comparable data were described in the study conducted by Lee and Finn (2007) [41].

In our study, lingonberry juice was noted as a source of glucosides, rutinoside of cyanidin, petunidin and pelargonidin. The most abundant anthocyanin detected in this juice was cyanidin-3-glucoside with a concentration of 0.605 ± 0.054 µg/mL. Other polyphenols in lingonberry juice were detected by LC-MS. These were mainly flavonols (kaempferol-3-glucoside, quercetin, quercetin-3-glucoside, quercetin-3-rhamnoside) as well as catechin, epicatechin, procyanidin dimer and procyanidin trimer. It is noteworthy that out of all the tested juices only the lingonberry contained proanthocyanidins. Reports have shown that proanthocyanidins in lingonberry and American cranberry are responsible for the health-promoting and antimicrobial activity of the juices [12].

The least varied composition of anthocyanins and other phenolic compounds detected by HPLC and LC-MS techniques was in the *Cornus mas* juice. Among anthocyanidins cyanidin-3-glucoside (0.280 ± 0.039 µg/mL), petunidin-3-glucoside (0.380 ± 0.052 µg/mL), cyanidin-3-robinobioside (0.321 ± 0.041 µg/mL) and pelargonidin-3-robinobioside (0.302 ± 0.022 µg/mL) were noted. Flavonoids identified in this juice with the use of liquid chromatography mass spectrometry included quercetin, quercetin-3-glucoside and myricetin-3-galactoside. It has been found that cornelian cherry contained quercetin and kaempferol, as well as cyanidin-3-galactoside, pelargonidin-3-glucoside, and pelargonidin-3-rutinoside. In the cornelian cherry fruits, cultivars of Bosnia and Herzegovina, the major component is peonidin-3-glucoside followed by cyanidin-3-galactoside [19]. Milenković-Andjelković (2015) described that the main anthocyanidin in cornelian cherry was pelargonidin-3-glucoside, which agrees with our results [43]. Taking into account the results of the above studies and results obtained in our case it can be stated that differences in fruit composition may occur due to genetic conditions of plants as well as climatic conditions in a given region. It has been described that genetic factors of the plant, as well as sun exposure, temperature, humidity, availability of nutrients and overall soil properties can influence the levels of particular anthocyanins and the content of polyphenols in general [23].

### 2.4. Bacterial Adhesion

**Figure 2:**
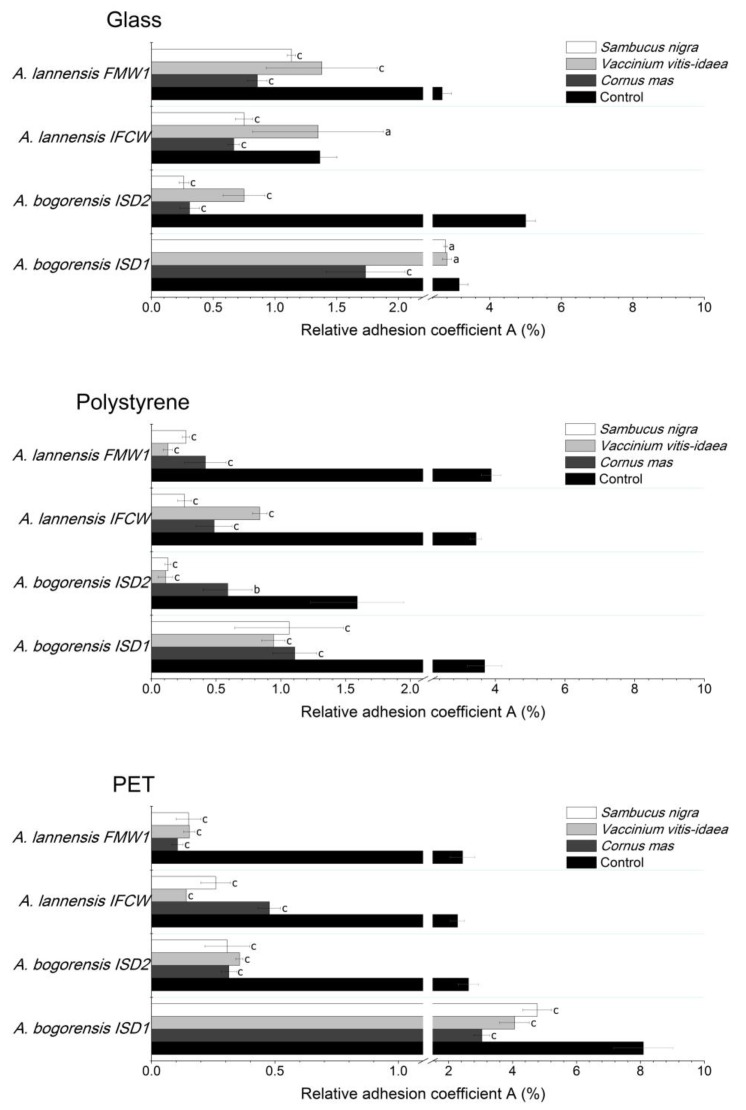
Adhesion of *Asaia* strains (*Asaia lannensis* and *Asaia bogorensis*) to glass, polystyrene and polyethylene terephthalate (PET) in minimal medium with addition of 10% elderberry, lingonberry and cornelian cherry. Results are expressed as relative adhesion coefficient A(%). Values are means of three determinations ± standard deviation. Values with the different letters are statistically different (*p* < 0.05). a—*p* ≥ 0.05; b—0.005 < *p* < 0.05; c—*p* < 0.005; The results were compared to those received for a control medium.

**Figure 3:**
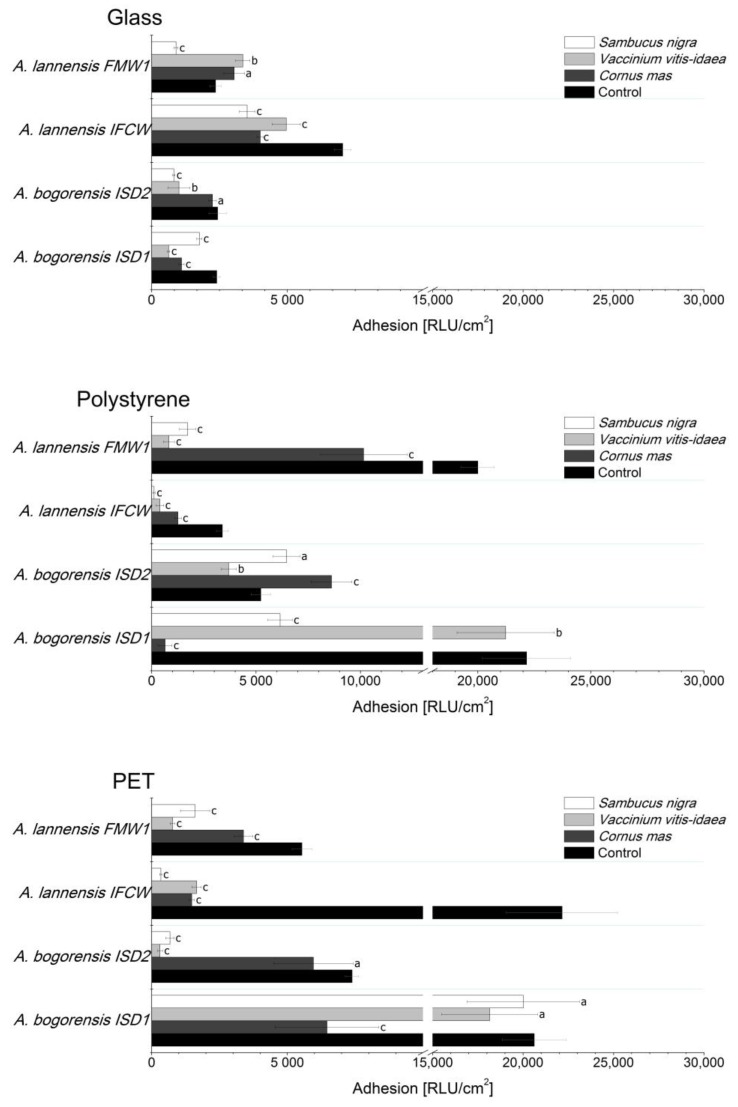
Adhesion of *Asaia* strains to glass, polystyrene and PET in minimal medium with addition of 10% elderberry, lingonberry and cornelian cherry. Results are expressed in relative light units (RLU)/cm^2^. Values are means of three determinations ± standard deviation. Values with the different letters are statistically different (*p* < 0.05). a—*p* ≥ 0.05; b—0.005 < *p* < 0.05; c—*p* < 0.005; The results were compared to those received for a control medium.

The determination of bacterial attachment and biofilm formation was conducted in the culture medium suitable for growth and adhesion of *Asaia* spp. strains isolated from commercial soft drinks [2,3]. As the parameter determining the affinity of bacterial cells to the surface, the relative adhesion coefficient A(%) was calculated. The intensity of biofilm formation was assessed by luminometric measurement and expressed in relative light units (RLUs). Bacterial cells and biofilm structures were observed using fluorescence microscopy with LIVE/DEAD BacLight Bacterial kit. The results of coefficient A(%) for adhesion of *Asaia lannensis* and *Asaia bogorensis* strains to glass (G), polystyrene (PS) and polyethylene terephthalate (PET) are presented in Figure 2, while Figure 3 shows the results of luminometric measurements. All of the tested *Asaia* strains showed strongest adhesion to polyethylene terephthalate material in minimal medium with the average value of 3.86 ± 0.46%; a slightly weaker effect was observed in the case of polystyrene (3.15 ± 0.32%) and glass (3.04 ± 0.23%). Results of adhesion coefficient A(%) were confirmed by the luminometric method in which relative lights units reached 13,923 ± 1360 RLU/cm^2^ (PET), 12,692 ± 855 RLU/cm^2^ (PS) and 3556 ± 241 RLU/cm^2^ (G) respectively. Comparison of results and obtained p values showed that these differences are statistically significant. Based on this information it can be stated that the adhesion of *Asaia* spp. are characterized by stronger adhesion to plastic materials.

The comparative results were obtained in previous studies conducted by Kręgiel (2013) [2]. It has been documented that the surface’s roughness and hydrophobicity significantly affects bacterial attachment and biofilm development. For instance, low surface energy promotes the adhesion of microorganisms. Polystyrene and polyethylene terephthalate used in our study are generally characterized by a lower surface energy than glass. However, the key factor affecting the adhesion is the environment. It has been noted that adhesion of *Asaia* spp. in media containing sucrose as the only carbon source is enhanced in comparison to environments with glucose or fructose content [3]. In addition, it is believed that modification of media composition through the use of antimicrobial substances that are safe to consumers’ health is the best strategy to prevent biofouling in soft drink technology.

Application of 10% lingonberry, cornelian cherry and elderberry juices as supplements to the minimal medium resulted in a significant decrease in the relative adhesion coefficient A(%) and luminometric measurement results. Slight decrease of A(%) in the medium containing lingonberry was observed only in the case of the *Asaia lannensis* IFCW strain. The strongest anti-adhesive properties were noted for elderberry juice, which inhibited the adhesion of *Asaia* spp. to the polystyrene carrier by 87% on average. Slightly weaker properties were noted for lingonberry juice (85%), and cornelian cherry (77%). Considering the species of tested bacteria, tested juices were characterized by a stronger anti-adhesive activity in relation to *A. lannensis*, inhibiting the attachment of the cells by 75%. The strongest inhibition was noted for *A. lannensis* FMW1 in the medium with lingonberry, from 3.88% to 0.12% (97%). Generally, elderberry and cornelian cherry juices showed comparable antiadhesive properties against the tested strains. Average results of the reduction of relative adhesion coefficient A(%) in these juices for all of the carriers were 74% and 73%, respectively, while in the case of lingonberry it was 67%. Luminometric results (RLU/cm^2^) confirmed significant reduction of *Asaia* spp. adhesion in the majority of tested juices. The most pronounced decrease in the results of luminometric measurements was noted for adhesion of *A. lannensis* IFCW in the medium with elderberry juice: from 22,153 RLU/cm^2^ to 339 RLU/cm^2^ (98%). Again, the used juices showed stronger activity against the *A. lannensis* causing a decrease in adhesion, by 60% on average. In general, *S. nigra* juice was characterized by the strongest anti-adhesive properties. The average reduction of adhesion (measured by the luminometric method) was 59%, while for *V. vitis-idaea* and *C. mas* it was 52% and 37%, respectively.

Obviously, the applied techniques of adhesion analysis involve measurement errors, but neither method is perfect. The techniques were selected by taking into account two important criteria: (1) complementary description of the effect of fruit juices on population and vitality of *Asaia* spp. cells attached to the surfaces; and (2) industrial applicability, where the bacteria produce biofilms on industrial lines, contaminate products and cause significant financial losses [3]. Generally, the plate count method allows for determination of culturable microorganisms, while the luminometric technique enables the estimation of total biological material on the abiotic surfaces. This method is based on ATP quantification and can be used to evaluate the total number of adhered cells, but also bacteria that are unable to grow and extracellular polymeric substances containing small amounts of ATP, as well as organic material from culture media. In addition, luminometric measurements may be influenced by bioactive compounds, such as (poly)phenolics, contained in the environment. Due to the mechanism of the luminometry measurement which is based on the enzymatic reaction of luciferin oxidation to oxyluciferin, the presence of antioxidants can influence the final results and may cause differences [44].

**Figure 4:**
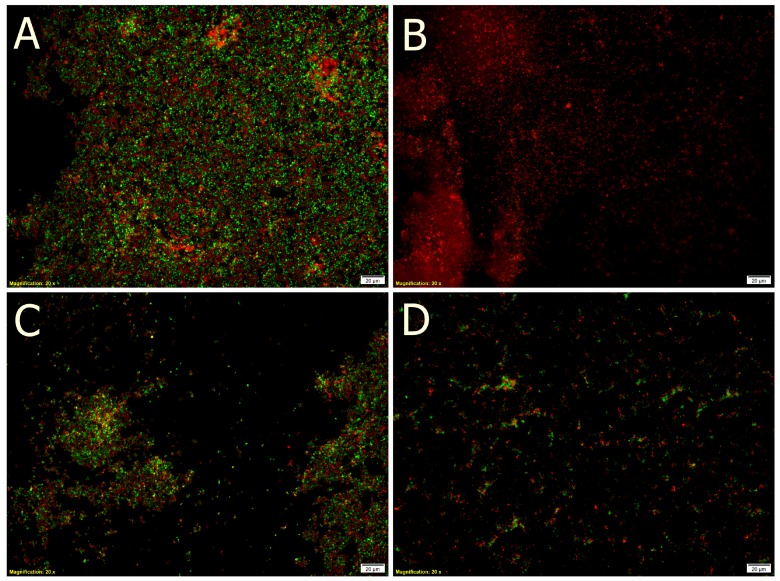
Microscopic observation of the biofilms formed in: (**A**) control (minimal medium); (**B**) medium with cornelian cherry juice; (**C**) medium with lingonberry juice; (**D**) medium with elderberry juice.

Comparison of the biofilm structures in the control medium to these with juices is shown in Figure 4. Microscopic analysis of the effect of tested juices on the adhesion abilities of *Asaia* strains showed that cornelian cherries only slightly affect the structure of the developed biofilm, but reduce the viability of the cells in the structures. Bacterial viability kits used in our study are a mixture of SYTO^®^ 9 (green-fluorescent nucleic acid) stain and propidium iodide (red-fluorescent nucleic acid) stain. Generally, SYTO^®^ 9 used alone labels all bacteria in the population, those with damaged membranes as well as those with intact membranes. On the other hand, propidium iodide exhibits activity only in relation to bacteria with damaged membranes. At the same time it causes a reduction in the SYTO stain. As a result, undamaged cells present green fluorescence while cells with damaged membranes are red.

Comparing biofilm images obtained for control (Figure 4A) and culture conducted with cornelian cherry juice (Figure 4B) we noted the reduction of viability of *Asaia bogorensis* ISD1 bacterial cells. At the same time, in the case of cultures conducted with lingonberry (Figure 4C) and elderberry (Figure 4D), significant changes in the structure of the biofilm were noted. In the case of *A. bogorensis* biofilm in the medium supplemented with elderberry juice, micro-colonies were observed. A similar effect was noted for lingonberry juice. Presumably, both the *S. nigra* as well as *V. vitis-idaea* are characterized by anti-adhesive activities, preventing cell adhesion to the surface, and consequently preventing the development of biofilm.

According to the literature, proanthocyanidins in cranberry and lingonberry juice are characterized by strong antiadhesive activities against uropathogenic *Escherichia coli* strains. It was documented that these compounds show urinary tract infection-preventive effect [12]. In addition, flavonoids from *V. vitis-idaea* showed strong activity against oral pathogens. It has been noted that flavan-3-ols and procyanidins dimers are active against biofilm formation of *Streptococcus mutans* and *Fusobacterium nucleatum* [45]. It has been described that the 10% addition of the elderberry extract decreased the growth of *Streptococcus pyogenes* and *Branhamella catarrhalis* (*Moraxella catarrhalis*) by 70% [46]. In addition, extract of elder fruits inhibits the growth of *Helicobacter pylori* by 20% [47]. On the other hand extracts from *Cornus mas* have been shown to possess strong antibacterial activity against both Gram-positive and Gram-negative bacteria: *Staphylococcus aureus* and *Pseudomonas aeruginosa* [48]. Epicatechin-(4β→8)-epicatechin-(4β→8,2β→*O*→7)-catechin also showed strong antimicrobial activity against *Porphyromonas gingivalis* and *Prevotella intermedia* [49]. Moreover, extracts containing tannins have been described as strong antibacterial agents against *Staphylococcus aureus*, *Helicobacter pylori*, *Clostridium perfringens*, *Bacillus cereus*, *Klebsiella* spp., and *Proteus* spp. [11]. It has been suggested that the inhibitory effect on the bacterial growth and adhesion may not result from the activity of simple phenolics compounds but occurs through the complex phenolic polymers. Generally, it is believed that several mechanisms are responsible for the antimicrobial properties of phenolic compounds: (1) destabilization and permeabilization of cytoplasmic membrane; (2) inhibition of extracellular microbial enzymes; (3) direct actions on microbial metabolism, and deprivation of the substrates; and (4) blocking of the microbial adhesins [32]. In addition, it has been noted that anthocyanins (pelargonidin, delphinidin, cyanidin, as well as cyanidin-3-glucoside) are characterized by growth inhibition of the DNA repair mutant strain of *E. coli*. Therefore, the antibacterial activity of these compounds can result from their reaction with DNA [11]. However, it is believed that antimicrobial activity of a fruit extracts is a synergistic effect of (poly)phenolic compounds. Our results confirmed this relationship. The average results of relative adhesion coefficient A(%) and luminometry (RLU/cm^2^) obtained in our study strongly correlate with the results of TPC tests. For A(%) method, the R coefficient amounted to 0.9228, while for RLU measurement the R was equal to 0.9641. Thus, it can be stated that the anti-adhesive properties of the tested juices depends on the content of polyphenols.

## 3. Materials and Methods

### 3.1. Plant Material

Cornelian cherry (*Cornus mas*), lingonberry (*Vaccinium vitis-idaea*) and elderberry (*Sambucus nigra*) fruits were collected fresh from local orchards and forests in central Poland in late July and early August 2016. Following this, fruits were washed with water, slightly dried, and frozen at −20 °C. The juice was obtained from defrosted fruits using a squeezer MES3000 (Bosch, Warsaw, Poland). The cloudy juice was passed through a 20-µm-pore-size filter paper (Whatman, Pittsburgh, PA, USA) once and then filtered and sterilized simultaneously with 0.45-µm-pore-size membranes (Merck-Millipore, Darmstadt, Germany).

### 3.2. Bacterial Cultures

Four strains of bacteria *Asaia* spp., isolated from fruit-flavoured mineral waters and isotonic drinks were used in the study—*Asaia bogorensis* ISD1 (GenBank KP234014), *A. bogorensis* ISD2 (GenBank KP234015), *A. lannensis* IFCW (GenBank KP234011) and *A. lannensis* FMW1 (GenBank HQ917850). These bacteria were identified using morphological, physiological and genetic methods and the nucleotide sequences of 16S rRNA were deposited in the GenBank (NCBI) [50]. Bacterial strains were deposited in the Pure Culture Collection of Industrial Microorganisms LOCK 105, at the Institute of Fermentation Technology and Microbiology, Lodz University of Technology (Poland).

### 3.3. Carriers

Bacterial adhesion was evaluated to plastics and glass used as packaging materials for soft drinks. For this purpose polystyrene (PS) (Coveris Rigid, Skierniewice, Poland) and polyethylene terephthalate (PET) (Coveris Rigid, Skierniewice, Poland) were used. The rectangular slides measuring 76 × 26 mm were sterilized in two stages: (1) carriers were kept in the 70% ethanol solution for 6 h; and then (2) they were placed in a laminar chamber (Telstar, Terrassa, Spain) and subjected to UV irradiation for 3 h. White glass slides (G) (Knittel Glass, Braunschweig, Germany) were used as the reference material. Tested plastics are certified by Polish National Institute of Public Health and intended for food contact.

### 3.4. Chemical Analysis of Juices

#### 3.4.1. Carbohydrates

The monosaccharide profiles of the tested juices were determined enzymatically using a UV spectrophotometer MULTISCAN GO (Thermo Fisher Scientific, Waltham, MA, USA) [51]. d-glucose and d-fructose content was determined in accordance with the procedures of the manufacturer of K-FRUGL assay kit (Megazyme, Bray, Ireland). The obtained results were calculated and expressed as grams of fructose or glucose per 100 mL of tested juice [g/100 mL].

#### 3.4.2. Total Phenolic Content (TPC)

Total phenolic content (TPC) was determined in accordance with the modified Folin-Ciocalteu method using a 6405 UV/VIS (ultraviolet–visible) spectrophotometer (Jenway, Stone, UK). Ten microliters of ten-folded juice and 100 µL of 10% (*v*/*v*) Folin-Ciocalteu’s reagent were mixed and incubated for 4 min at room temperature. Subsequently 100 µL of 7% (*w*/*v*) sodium carbonate and 40 µL of distilled water were added. After the incubation of the mixture in darkness, at room temperature for 1.5 h, absorbance was measured at 765 nm. Simultaneously, a standard curve of gallic acid was prepared using concentrations from 0 to 250 mg/L, and the correlation coefficient was 0.9998. Total phenolic content was calculated as mg of gallic acid equivalents (GAE) per mL of sample (mg GAE/mL).

#### 3.4.3. Total Antioxidant Capacity (DPPH)

The total antioxidant capacity of juices was determined spectrophotometrically (Jenway, Stone, UK). DPPH was freshly prepared in 96% methanol. The stock solution was prepared by dissolving 24 mg of 1,1-diphenyl-2-picrylhydrazyl (DPPH) with 100 mL of methanol. The working solution was obtained by dilution of the stock solution with methanol to obtain an absorbance of approximately 1.00 ± 0.05 at 515 nm. A total of 150 µL of properly diluted (five-fold dilutions) sample juice was added to 2.85 mL of DPPH. The solution was incubated in darkness, at room temperature for 1 h. The results were expressed as IC_50_ (g/mL)—the concentration of the tested juice leading to 50% reduction of the initial DPPH concentration. Lower absorbance of the reaction mixture indicated higher free radical-scavenging activity [52].

#### 3.4.4. Ferric-Reducing Antioxidant Power (FRAP)

The ferric-reducing antioxidant power (FRAP) of the berries was tested following the assay of Oyaizu (1986) [53]. The sample of tested juice was diluted with sterile distilled water to obtain a series of five-fold dilutions. Then, 0.5 mL of the proper dilution was added to 2.5 mL of 0.2 M phosphate buffer, pH 6.6 and 1% of potassium iron (III) hexacyanoferrate (II). In the control sample, distilled water was used instead of juice. Thereafter, the samples were incubated for 20 min at 50 °C, then immediately cooled and treated with 10% trichloroacetic acid (TCA). Then, 2.5 mL of the supernatant was transferred to a new test tube with 2.5 mL of sterile distilled water and 1 mL of 0.1% (*w*/*v*) iron (III) chloride hexahydrate. The reducing power was determined spectrophotometrically (Jenway, Stone, UK) at 700 nm. The results were calculated and expressed as IC_50_ [g/mL].

#### 3.4.5. Phenolic Compounds

Before chromatographic analysis the juices were filtered with 0.45-µm-pore-size membranes (Merck-Millipore, Darmstadt, Germany). The phenolic compounds contained in tested juices were characterized using HPLC with a diode array detector (DAD) (Finnigan Surveyor-PDA Plus detector, Thermo Fisher Scientific, Waltham, MA, USA) and ChromQuest 5.0 chromatography software (Thermo Fisher Scientific, Waltham, MA, USA). Separation of anthocyanins using the HPLC method was achieved on a Lichrospher RP 18-5 (250 mm by 4.6 mm, 5 µm packing; Hichrom, Reading, UK). The elution conditions were as follows: flow rate of 0.8 mL/min; oven temperature of 25 °C; solvent A (5% (*v*/*v*) formic acid), and solvent B (95% (*v*/*v*) acetonitrile). Elution began with 3% solvent B for 2 min, then 3% to 15% solvent B for 13 min, 15% to 18% solvent B for 9 min, 18% to 25% solvent B for 31 min, and 25% to 30% solvent B for 5 min, followed by washing and re-equilibration of the column. The injection volume for all samples was 50 µL. Detection was conducted at 520 nm [3]. Individual anthocyanin contents were determined according to the linear calibration curve (R = 0.9947) and expressed as µg of cyanidin-3-glucoside per mL of sample.

Other phenolic compounds were also analyzed using the HPLC method described by Mišan et al. (2011) [54]. This analysis was performed by using an Agilent 1200 series (Agilent Technologies, Santa Clara, CA, USA) liquid chromatograph equipped with a diode array detector (DAD), a binary pump, an online vacuum degasser, Chemstation Software (Agilent Technologies, Santa Clara, CA, USA), an autosampler and a column (4.6 mm by 50 mm, 1.8 μm packing, Agilent Technologies, Eclipse XDB-C18), at a flow rate of 1 mL/min. Solvent gradient was performed by varying the proportion of solvent A (methanol) to solvent B (1% (*v*/*v*) formic acid in water). The elution conditions were as follows: initial 10% solvent A (methanol); 0–10 min, 10–25% solvent A; 10–20 min, 25–60% solvent A; and 20–30 min, 60–70% solvent A. The injection was done automatically using autosampler, and the volume of the tested sample and standards was 5 μL. The spectra were recorded within 60 min in the range 210–400 nm and chromatograms plotted at 280, 330 and 350 nm. The content of the phenolic compounds was determined according to its calibration curve and expressed as µg per mL of sample. Calibration curves were plotted on the basis of five calibration points and the correlation coefficients were calculated. For all investigated compounds the correlation coefficient was higher than 0.9995.

Polyphenols were characterized using a LC-MS method. Samples were injected onto an HPLC column coupled online to an LTQ Velos mass spectrometer (Thermo Fisher Scientific, Waltham, MA, USA). Chromatographic separation was achieved with a Hypersil GOLD column (1.9 µm, 150 mm by 4.6 mm; Thermo Fisher Scientific, Waltham, MA, USA) operated at 45 °C. The mobile phase consisted of solvent A (1 mL of formic acid in 1 L of deionized water) and solvent B (95% (*v*/*v*) acetonitrile). The elution began with 96% to 85% solvent A for 8 min, then 85% to 82% solvent A for 12 min, 82% to 60% solvent A for 40 min, 60% to 50% solvent A for 4 min and then 3 min, and 50% to 96% solvent A for 2 min, followed by washing and re-equilibration of the column. Mass spectra were recorded within 60 min. The injection volume was 10 µL. The flow rate was set at 220 µL/min. Electrospray ionization (ESI) mass spectrometry was performed using the LTQ Velos MS (Thermo Fisher Scientific, Waltham, MA, USA) equipped with a heated electrospray ionization interface and controlled by Xcalibur software (Thermo Fisher Scientific, Waltham, MA, USA). Mass spectra were acquired in negative mode over the *m*/*z* range of 120 to 1000. The ionization spray voltage was 4 kV. The sheath gas flow rate was 25 mL/min, and auxiliary gas flow rate was 10 mL/min. The temperatures of source and desolvation were 350 °C and 280 °C, respectively [55].

### 3.5. Microbiological Analysis

#### 3.5.1. Culture Media and Growth Conditions

Bacterial growth and adhesion were investigated in liquid minimal medium (2% sucrose (*w*/*v*), 0.3% (NH_4_)_2_PO_4_ (*w*/*v*), 0.3% KH_2_PO_4_ (*w*/*v*), 0.3% MgSO_4_ × 7H_2_O (*w*/*v*), 0.05% (*w*/*v*) yeast extract, and pH 5.8 ± 0.05) [3]. Sterile minimal medium (19.8 mL) was poured aseptically into 25 mL Erlenmeyer flasks, then sterile carriers—being surfaces for bacterial adhesion—were placed vertically into a liquid in such a way that half of the carrier was immersed in the medium, and the other part was above the liquid. Immediately after the preparation of fruit juices, they were added to the culture media to the final concentration of 10% (*v*/*v*). The addition of these juices caused the decrease in pH values. For minimal medium with *C. mas* juice the value was 4.30 ± 0.05, while for *V. vitis-idaea* and *S. nigra* the pH values were 4.45 ± 0.05 and 4.50 ± 0.05, respectively. Culture media were inoculated with standardized bacterial suspensions in order to obtain bacterial cell concentration of 10^5^–10^6^ colony-forming units per mL (CFU/mL). Bacterial cultures were incubated for 6 days at 25 °C.

#### 3.5.2. Bacterial Adhesion

Determination of the number of bacteria in the liquid and those attached to the surface was conducted using the plate count method with CG agar medium (2% (*w*/*v*) glucose, 0.3% (*w*/*v*) peptone, 0.3% (*w*/*v*) yeast extract, 0.7% (*w*/*v*) CaCO_3_, and 2% (*w*/*v*) agar). According to our previous studies, this medium is suitable for the growth of *Asaia* strains [6,50]. In order to determine the level of bacterial adhesion on abiotic surfaces, two methods—plate count and luminometry—were used. For this purpose the carrier was removed from the medium, washed with sterile distilled water and then swabbed with ATP-free, HY-LiTE^®^ sampling pens (Merck Millipore, Germany). The obtained results were converted to RLU per cm^2^ of the carrier. In order to determine the number of viable bacterial cells attached to the tested surface, the carrier was removed from the medium, rinsed with sterile distilled water and then swabbed. Then, the swab was transferred into 0.85% (*w*/*v*) sodium chloride with 0.1% (*v*/*v*) Tween 80, vortexed vigorously, diluted and transferred onto GC agar medium. Inoculated plates were incubated at 25 °C for 96 h, and the characteristic pink-pale colonies of *Asaia* spp. were counted. The results were expressed as CFU per cm^2^ of the carrier. The number of planktonic cells was also determined by plate count method and the results were presented as CFU per mL of culture medium. Based on the results obtained for both adhered and planktonic cells, the level of bacterial adhesion as the relative adhesion coefficient A(%) was calculated according to the formula described by Kręgiel (2013) [2].

#### 3.5.3. Fluorescent Microscopy

Visualization of bacterial biofilms was performed by fluorescence staining using a LIVE/DEAD™ BacLight™ Bacterial Viability Kit (Thermo Fisher Scientific, Waltham, MA, USA). The kit contains two dyes: SYTO^®^ 9 (green-fluorescent nucleic acid) stain and propidium iodide (red-fluorescent nucleic acid stain). Preparation of the staining solution was carried out in accordance with the manufacturers procedure, mixing the dyes in a ratio of 1:1. Biofilm was gently washed with phosphate buffered saline (PBS) solution, and then the entire surface was covered with a staining solution. The sample was incubated in darkness for 20 min at 30 °C. Images of the cells were taken using fluorescent microscope OLYMPUS BX53 equipped with filters with excitation wavelength ranging from 470 to 630 nm, and a high-resolution digital color camera (Olympus, Tokyo, Japan).

### 3.6. Statistics

Three independent experiments were performed, and from the obtained data, means with standard deviations were calculated. Statistical differences between the obtained adhesion results were compared using a one-way repeated measures analysis of variance (ANOVA; OriginPro 9.2.214, OriginLab Corp., Northampton, MA, USA). Statistical significance was set at the level of 5% (*p* < 0.05). In addition, correlation coefficients between results of DPPH, FRAP, TPC and those obtained for adhesion analysis were calculated. The chemical compositions of the tested fruit juices were compared using principal components analysis (PCA) using XLSTAT 2017 (Addinsoft, New York, NY, USA), complete statistical add-in for Microsoft Excel (Microsoft, Waszyngton, WA, USA). The outcomes from LC-MS were displayed as binary data (0 or 1) depending on whether a component was absent or present in the plant extract, while the outcomes from HPLC were displayed as a concentration of the specific component.

## 4. Conclusions

We have shown that some little-known, edible European fruits may present promising sources for the beverage industry, not only because of their strong antioxidant properties and high content of phenolic compounds. These juices may be a valuable supplement for functional beverages, inhibiting bacterial adhesion on abiotic surfaces. Therefore, multi-component fruit juices recognized by folk medicine may be used as components of modern functional drinks to improve their microbial stability. Further studies on active components based on novel strategies by computational techniques, chemistry, and cell-based models are necessary. While the discovery and development of natural products represents a complex endeavor demanding a highly integrated interdisciplinary approach, the research trends clearly indicate that wild edible fruits may be among the most important sources of new functional foods in the future.

## Figures and Tables

**Table 1:** Sugar content and antioxidant properties of investigated fruit juices.

Berry Juice	Sugar Content (g/100 mL)		TPC (mg GAE/mL)	Antioxidant Activity	
	Fructose	Glucose		DPPH IC_50_ (g/mL)	FRAP IC_50_ (g/mL)
Cornelian cherry (*Cornus mas*)	5.56 ± 0.061	2.97 ± 0.046	2.33 ± 0.013	0.045 ± 0.001	0.042 ± 0.001
Lingonberry (*Vaccinium vitis-idaea*)	3.89 ± 0.043 ^c^	4.54 ± 0.071 ^c^	4.87 ± 0.044 ^c^	0.054 ± 0.002 ^c^	0.030 ± 0.002 ^c^
Elderberry (*Sambucus nigra*)	3.29 ± 0.015 ^c^	3.19 ± 0.022 ^b^	8.02 ± 0.027 ^c^	0.072 ± 0.001 ^c^	0.021 ± 0.001 ^c^

GAE—gallic acid equivalents; TPC—total polyphenol content; DPPH—2,2-diphenyl-1-picrylhydrazyl; FRAP—ferric-reducing antioxidant power; IC_50_—inhibitory concentration. Values are means of three determinations ± standard deviation. Values in the same column with the different superscript lowercase letters are statistically different (*p* < 0.05). ^b^—0.005 < *p* < 0.05; ^c^—*p* < 0.005; The results were compared to those received for *C. mas*.

**Table 2:** Phenolic profiles of investigated berry juices.

Proposed Molecule	Concentration (µg/mL)		
	*Cornus mas*	*Vaccinium vitis-idaea*	*Sambucus nigra*
Caffeic acid	nd	nd	2.603 ± 0.313 ^d^
Cinnamic acid	0.143 ± 0.011	0.191 ± 0.014 ^b^	nd
Gallic acid	2.025 ± 0.314	0.071 ± 0.009 ^c^	0.286 ± 0.082 ^c^
Protocatechuic acid	0.379 ± 0.271	0.497 ± 0.087 ^a^	0.550 ± 0.057 ^a^
*p*-coumaric acid	0.108 ± 0.048	0.179 ± 0.052 ^a^	nd
Rosmarinic acid	0.128 ± 0.062	0.128 ± 0.037 ^a^	0.128 ± 0.019 ^a^
4-hydroxybenzoic acid	nd	0.150 ± 0.074 ^a^	0.265 ± 0.096 ^a^
Catechin	nd	0.662 ± 0.121 ^a^	0.918 ± 0.107 ^a^
Epicatechin	nd	0.304 ± 0.082 ^d^	nd
Rutin	nd	nd	1.321 ± 0.307 ^d^
Delphinidin-3-glucoside	nd	nd	2.057 ± 0.371 ^d^
Cyanidin-3-sambubioside-5-glucoside	nd	nd	2.260 ± 0.219 ^d^
Cyanidin-3-glucoside	0.280 ± 0.039	0.605 ± 0.054 ^c^	3.738 ± 0.147 ^c^
Cyanidin-3-sambubioside	nd	nd	3.143 ± 0.262 ^d^
Cyanidin-3-robinobioside	0.321 ± 0.041	nd	nd
Petunidin-3-galactoside	nd	0.320 ± 0.057 ^d^	nd
Petunidin-3-glucoside	nd	0.528 ± 0.052 ^d^	nd
Pelargonidin-3-glucoside	0.380 ± 0.052	0.359 ± 0.063 ^a^	nd
Pelargonidin-3-rutinoside	nd	0.344 ± 0.074 ^d^	nd
Pelargonidin-3-robinobioside	0.302 ± 0.022	nd	nd

Values are means of three determinations ± standard deviation. Values in the same row with the different superscript lowercase letters are statistically different (*p* < 0.05). ^a^—*p* ≥ 0.05; ^b^—0.005 < *p* < 0.05; ^c^—*p* < 0.005; ^d^—not compared; The results were compared to those received for *C. mas*. Anthocyanin contents were expressed as µg of cyanidin-3-glucoside per one mL. nd—not detected

**Table 3:** Major polyphenolic compounds present in the tested juices, using the LC-MS method.

Aglycone Class	Proposed Molecule	λmax (nm)	[M − H]^−^	MS^2^	*Cornus mas*	*Vaccinium vitis-idaea*	*Sambucus nigra*
**Phenolic acids**	Caffeic	279	179	135	−	−	+
	Caffeic acid derivative	234, 279	341	177, 195	−	−	+
	Caffeoyl hexoside	231, 282	341	179	−	+	−
	Chlorogenic	295, 323	353	191	−	+	+
	Neochlorogenic	323	353	179, 191	−	+	+
	Ferulic	237, 323	193	149, 173	+	−	−
	Gallic	237, 276	205	111, 125, 173	+	+	+
	Quinic	235, 284	191	111, 173	−	+	−
**Flavonols**	Kaempferol	239, 279, 325	285	213, 257	−	−	+
	Kaempferol-3-glucoside	263, 344	447	255, 284, 327	−	+	+
	Kaempferol-3-rutinoside	265, 342	593	285	−	−	+
	Quercetin	235, 279, 341	301	229, 255	+	+	+
	Quercetin-3-glucoside	257, 353	463	301	+	+	+
	Quercetin-3-rhamnoside	257, 349	447	301	−	+	−
	Quercetin-3-*O*-rutinoside (Rutin)	256, 350	609	301	-	-	+
	Myricetin-3-galactoside	238, 278	491	317	+	−	−
**Anthocyanins**	Delphinidin-3-glucoside	276	463	301	−	+	+
	Cyanidin-3-glucoside	282	449	287	+	+	+
	Petunidin-3-glucoside	236, 269	479	317	+	+	−
	Peonidin-3-galactoside	235, 280	465	301	−	−	+
	Pelargonidin-3-robinobioside	271	577	431, 269	+	−	−
	Cyanidin-3-samburoside	279	581	449, 287	−	−	+
	Cyanidin-3-robinobioside	280	593	447, 285	+	−	−
**Flavanols**	Catechin	233, 280	289	205, 245	−	+	−
	Epicatechin	231, 281	289	205, 245	−	+	−
**Proantho-cyanidins**	Procyanidin dimer	281	575	425, 407	-	+	−
	Procyanidin trimer	277	863	575	−	+	−
**Others**	Coumaroyl iridoid	238, 282	366	309	−	+	−
	Cornuside	242, 274	541	169, 347	+	−	−
	Loganic	239, 279	375	213, 169	+	−	−
	Loganic acid	239, 279	375	213, 169	+	−	−

−—not detected; +—present in the sample.

## References

[1] Lia R., Kudab T., Yanoa T. (2014). Effect of food residues on efficiency of surfactant disinfectants against food related pathogens adhered on polystyrene and ceramic surfaces. LWT Food Sci. Technol..

[2] Kręgiel D. (2013). Attachment of *Asaia lannensis* to materials commonly used in beverage industry. Food Control.

[3] Antolak H., Czyżowska A., Kręgiel D. (2016). Black currant (*Ribes nigrum* L.) and bilberry (*Vaccinium myrtillus* L.) fruit juices inhibit adhesion of *Asaia* spp.. BioMed Res. Int..

[4] Rushton L., Sass A., Baldwin A., Dowson C.G., Donoghue D., Mahenthiralingam E. (2013). Key role for efflux in the preservative susceptibility and adaptive resistance of *Burkholderia cepacia* complex bacteria. Antimicrob. Agents Chemother..

[5] Charlebois A., Jacques M., Boulianne M., Archambault M. (2017). Tolerance of *Clostridium perfringens* biofilms to disinfectants commonly used in the food industry. Food Microbiol..

[6] Antolak H., Czyżowska A., Kręgiel D. (2017). Antibacterial and antiadhesive activities of extracts from edible plants against soft drink spoilage by *Asaia* spp.. J. Food Prot..

[7] Borlinghaus J., Albrecht F., Gruhlke M.C.H., Nwachukwu I.D., Slusarenko A.J. (2014). Allicin: Chemistry and biological properties. Molecules.

[8] Newman D.J., Cragg G.M. (2016). Natural products as sources of new drugs from 1981 to 2014. J. Nat. Prod..

[9] Atanasov A.G., Waltenberger B., Pferschy-Wenzig E.M., Linder T., Wawrosch C., Uhrin P., Temml V., Wang L., Schwaiger S., Heiss E.H. (2015). Discovery and resupply of pharmacologically active plant-derived natural products: A review. Biotechnol. Adv..

[10] Lipińska L., Klewicka E., Sójka M. (2014). Structure, occurrence and biological activity of ellagitannins: A general review. Acta Sci. Pol. Technol. Aliment..

[11] Nohynek L.J., Alakomi H.L., Kähkönen M.P., Heinonen M., Helander I.M., Oksman-Caldentey K.M., Puupponen-Pimiä R.H. (2006). Berry phenolics: Antimicrobial properties and mechanisms of action against severe human pathogens. Nutr. Cancer.

[12] Howell A., Souza D., Roller M., Fromentin E. (2015). Comparison of the anti-adhesion activity of three different cranberry extracts on uropathogenic P-fimbriated *Escherichia coli*: A randomized, double-blind, placebo controlled, ex vivo, acute study. Nat. Prod. Commun..

[13] Gasparrini M., Forbes-Hernandez T.Y., Giampieri F., Afrin S., Alvarez-Suarez J.M., Mazzoni L., Mezzetti B., Quiles J.L., Battino M. (2017). Anti-inflammatory effect of strawberry extract against LPS-induced stress in RAW 264.7 macrophages. Food Chem. Toxicol..

[14] Afrin S., Gasparrini M., Forbes-Hernandez T.Y., Reboredo-Rodriguez P., Mezzetti B., Varela-López A., Giampieri F., Battino M. (2016). Promising health benefits of the strawberry: A focus on clinical studies. J. Agric. Food Chem..

[15] Veberic R., Slatnar A., Bizjak J., Stampar F., Mikulic-Petkovsek M. (2015). Anthocyanin composition of different wild and cultivated berry species. LWT Food Sci. Technol..

[16] Szajdek A., Borowska E.J. (2008). Bioactive compounds and health-promoting properties of berry fruits: A review. Plant. Foods Hum. Nutr..

[17] Forbes-Hernandez T.Y., Gasparrini M., Afrin S., Bompadre S., Mezzetti B., Quiles J.L., Giampieri F., Battino M. (2016). The healthy effects of strawberry polyphenols: Which strategy behind antioxidant capacity?. Crit. Rev. Food Sci. Nutr..

[18] Sidor A., Gramza-Michałowska A. (2015). Advanced research on the antioxidant and health benefit of elderberry (*Sambucus nigra*) in food—A review. J. Funct. Foods.

[19] Drkenda P., Spahic A., Begic-Akagic A., Gasi F., Vranac A., Hudina M., Blanke M. (2014). Pomological characteristics of some autochthonous genotypes of cornelian cherry (*Cornus mas* L.) in Bosnia and Herzegovina. Erwerbs-Obstbau.

[20] Tolmacheva A.A., Rogozhin E.A., Deryabin D.G. (2014). Antibacterial and quorum sensing regulatory activities of some traditional Eastern-European medicinal plants. Acta Pharm..

[21] Veberic R., Jakopic J., Stampar F., Schmitzer V. (2009). European elderberry (*Sambucus nigra* L.) rich in sugars, organic acids, anthocyanins and selected polyphenols. Food Chem..

[22] Visti A., Viljakainen S., Laakso S. (2003). Preparation of fermentable lingonberry juice through removal of benzoic acid by *Saccharomyces cerevisiae* yeast. Food Res. Int..

[23] Cheng G., He Y.-N., Yue T.-X., Wang J., Zhang Z.-W. (2014). Effects of climatic conditions and soil properties on cabernet sauvignon berry growth and anthocyanin profiles. Molecules.

[24] Prior R.L., Sintara M., Chang T. (2016). Multi-radical (ORACMR5) antioxidant capacity of selected berries and effects of food processing. J. Berry Res..

[25] Viskelis P., Rubinskienė M., Bobinaitė R., Dambrauskienė E. (2010). Bioactive compounds and antioxidant activity of small fruits in Lithuania. J. Food Agric. Environ..

[26] Jakobek L., Šeruga M., Medvidović-Kosamović M., Novak I. (2007). Anthocyanin content and antioxidant activity of various red fruit juices. Deutsch. Lebensm. Rundshau.

[27] Ersoy N., Bagci Y., Gok V. (2011). Antioxidant properties of 12 cornelian cherry fruit types (*Cornus mas* L.) selected from Turkey. Sci. Res. Essays.

[28] Georgieva M., Badjakov I., Dincheva I., Yancheva S., Kondakova V. (2016). In vitro propagation of wild Bulgarian small berry fruits (bilberry, lingonberry, raspberry and strawberry). Bulg. J. Agric. Sci..

[29] Tarko T., Duda-Chodak A., Semik D., Nycz M. (2015). The use of fruit extracts for production of beverages with high antioxidative activity. Potravin. Slovak J. Food Sci..

[30] Šamec D., Piljac-Žegarac J. (2011). Postharvest stability of antioxidant compounds in hawthorn and cornelian cherries at room and refrigerator temperatures—Comparison with blackberries, white and red grapes. Sci. Hort..

[31] Moldovan B., Filip A., Clichici S., Suharoschi R., Bolfa P., David L. (2016). Antioxidant activity of Cornelian cherry (*Cornus mas* L.) and the in vivo evaluation of its anti-inflammatory effects. J. Funct. Foods.

[32] Nile S.H., Park S.W. (2014). Edible berries: Bioactive components and their effect on human health. Nutrition.

[33] Silva P., Ferreira S., Nunes F.M. (2017). Elderberry (*Sambucus nigra* L.) by-products a source of anthocyanins and antioxidant polyphenols. Ind. Crops Prod..

[34] Kucharska A.Z., Sokół-Łętowska A., Oszmiański J., Piórecki N., Fecka I. (2017). Iridoids, phenolic compounds and antioxidant activity of edible honeysuckle berries (*Lonicera caerulea* var. *kamtschatica* Sevast.). Molecules.

[35] Hosseini-Behesht E., Lund S.T., Kitts D.D. (2012). Characterization of antioxidant capacity from fruits with distinct anthocyanin biosynthetic pathways. J. Nutr. Food Sci..

[36] Deng S., West B.J., Jensen C.J. (2013). UPLC-TOF-MS characterization and identification of bioactive iridoids in *Cornus mas* fruit. J. Anal. Methods Chem..

[37] Radovanović B.C., Anđelković A.S.M., Radovanović A.B., Anđelković M.Z. (2013). Antioxidant and antimicrobial activity of polyphenol extracts from wild berry fruits grown in southeast Serbia. Trop. J. Pharm. Res..

[38] Häkkinen S., Heinonen M., Kärenlampi S., Mykkänen H., Ruuskanen J., Törrönen R. (1999). Screening of selected flavonoids and phenolic acids in 19 berries. Food Res. Int..

[39] Mattila P., Hellström J., Törrönen R. (2006). Phenolic acids in berries, fruits, and beverages. J. Agric. Food Chem..

[40] Tarko T., Duda-Chodak A., Wajda Ł., Satora P., Sroka P., Emik-Szczurak D. (2017). Application of principal component analysis for optimization of polyphenol extraction from alternative plant sources. J. Food Nutr. Res..

[41] Lee J., Finn E.F. (2007). Anthocyanins and other polyphenolics in American elderberry (*Sambucus canadensis*) and European elderberry (*S. nigra*) cultivars. J. Sci. Food Agric..

[42] Mikulic-Petkovsek M., Ivancic A., Todorovic B., Veberic R., Stampar F. (2015). Fruit phenolic composition of different elderberry species and hybrids. J. Food Sci..

[43] Milenković-Andjelković A.S., Andjelković M.Z., Radovanović A.N., Radovanović B.C., Nikolić V. (2015). Phenol composition, DPPH radical scavenging and antimicrobial activity of Cornelian cherry (*Cornus mas*) fruit and leaf extracts. Hem. Ind..

[44] He Q., Lv Y., Yao K. (2007). Effects of tea polyphenols on the activities of α-amylase, pepsin, trypsin and lipase. Food Chem..

[45] Riihinen K.R., Ou Z.M., Gödecke T., Lankin D.C., Pauli G.F., Wu C.D. (2014). The antibiofilm activity of lingonberry flavonoids against oral pathogens is a case connected to residual complexity. Fitoterapia.

[46] Krawitz C., Mraheil M.A., Stein M., Imirzalioglu C., Domann E., Pleschka S., Hain T. (2011). Inhibitory activity of a standardized elderberry liquid extract against clinically-relevant human respiratory bacterial pathogens and influenza A and B viruses. BMC Complement. Altern. Med..

[47] Chatterjee A., Yasmin T., Bagchi D., Stohs S.J. (2004). Inhibition of *Helicobacter pylori* in vitro by various berry extracts, with enhanced susceptibility to clarithromycin. Mol. Cell. Biochem..

[48] Kyriakopoulos A.M., Dinda B. (2015). *Cornus mas* (*Linnaeus*) novel devised medicinal preparations: bactericidal effect against *Staphylococcus aureus* and *Pseudomonas aeruginosa*. Molecules.

[49] Ho K.Y., Tsai C.C., Huang J.S., Chen C.P., Lin T.C., Lin C.C. (2001). Antimicrobial activity of tannin components from *Vaccinium vitis-idaea* L.. J. Pharm Pharmacol..

[50] Kręgiel D., Otlewska A., Antolak H. (2014). Attachment of *Asaia bogorensis* originating in fruit-flavored water to packaging materials. BioMed Res. Int..

[51] Berłowska J., Cieciura W., Borowski S., Dudkiewicz M., Binczarski M., Witonska I., Otlewska A., Kregiel D. (2016). Simultaneous saccharification and fermentation of sugar beet pulp with mixed bacterial cultures for lactic acid and propylene glycol production. Molecules.

[52] Mišan A.C., Sakač M., Medić Đ., Tadić V., Marković G., Gyura J., Pagano E., Izzo A.A., Borrelli F., Šarić B. (2016). Antioxidant and physicochemical properties of hydrogen peroxide-treated sugar beet dietary fibre. Phytother. Res..

[53] Oyaizu M. (1986). Studies on products of browning reaction: antioxidative activities of products of browning reaction prepared from glucosamine. Jpn. J. Nutr..

[54] Mišan A.C., Mimica-Dukić N.M., Mandić A.I., Sakač M.B., Milovanović I.J., Sedej I.J. (2011). Development of a rapid resolution HPLC method for the separation and determination of 17 phenolic compounds in crude plant extracts. Cent. Eur. J. Chem..

[55] Brodowska A.J., Śmigielski K., Nowak A., Czyżowska A., Otlewska A. (2015). The impact of ozone treatment in dynamic bed parameters on changes in biologically active substances of juniper berries. PLoS ONE.

